# Assessment by Optical Coherence Tomography of Short-Term Changes in IOP-Related Structures Caused by Wearing Scleral Lenses

**DOI:** 10.3390/jcm12144792

**Published:** 2023-07-20

**Authors:** Juan Queiruga-Piñeiro, Alberto Barros, Javier Lozano-Sanroma, Andrés Fernández-Vega Cueto, Ignacio Rodríguez-Uña, Jesús Merayo-LLoves

**Affiliations:** 1 Instituto Universitario Fernández-Vega, Fundación de Investigación Oftalmológica, Universidad de Oviedo, 33012 Oviedo, Spain; alberto.barros@fernandez-vega.com (A.B.); javilo@fernandez-vega.com (J.L.-S.); afvc@fernandez-vega.com (A.F.-V.C.); merayo@fio.as (J.M.-L.); 2 Instituto de Investigación Sanitaria del Principado de Asturias (ISPA), 33011 Oviedo, Spain; 3 Department of Surgery and Medical-Surgical Specialities, Universidad de Oviedo, 33006 Oviedo, Spain

**Keywords:** scleral lenses, intraocular pressure, iridocorneal angle, Schlemm’s canal, optic nerve head, optical coherence tomography, transpalpebral tonometry, Perkins applanation tonometry

## Abstract

Background: The mechanism that could increase intraocular pressure (IOP) during scleral lens (SL) wear is not fully understood, although it may be related to compression of the landing zone on structures involved in aqueous humor drainage. Methods: Thirty healthy subjects were fitted with two SLs of different sizes (L1 = 15.8 mm, L2 = 16.8 mm) for 2 h in the right eye and left eye as a control. Central corneal thickness (CCT), parameters of iridocorneal angle (ICA), Schlemm’s canal (SC), and optic nerve head were measured before and after wearing both SLs. IOP was measured with a Perkins applanation tonometer before and after lens removal and with a transpalpebral tonometer before, during (0 h, 1 h, and 2 h), and after lens wear. Results: CCT increased after wearing L1 (8.10 ± 4.21 µm; *p* < 0.01) and L2 (9.17 ± 4.41 µm; *p* < 0.01). After L1 removal, the ICA parameters decreased significantly (*p* < 0.05). With L2 removal, nasal and temporal SC area and length were reduced (*p* < 0.05). An increased IOP with transpalpebral tonometry was observed at 2 h of wearing L1 (2.55 ± 2.04 mmHg; *p* < 0.01) and L2 (2.53 ± 2.22 mmHg; *p* < 0.01), as well as an increased IOP with Perkins applanation tonometry after wearing L1 (0.43 ± 1.07 mmHg; *p* = 0.02). Conclusions: In the short term, SL resulted in a slight increase in IOP in addition to small changes in ICA and SC parameters, although it did not seem to be clinically relevant in healthy subjects.

## 1. Introduction

Multiple studies demonstrated an increase in intraocular pressure (IOP) with scleral lens (SL) wear [1,2,3,4,5,6]. This increase varies according to the tonometer used [2,3,6] and the wearing time [1,2,6,7]. The transpalpebral tonometer (TT) is one of the few tonometers designed to perform IOP measurements with SLs in situ [1,2,4,8]. The largest increases in IOP, between 4.4 and 5.5 mmHg, have been obtained with this instrument during the wearing of SLs [1,2], but it has shown poor agreement with Goldmann applanation tonometry (GAT), considered the gold standard [9,10,11]. The GAT has also observed a slight increase in IOP after lens removal, around 1 mmHg, because measurements cannot be made during SL wear [4,6]. The pneumotonometer presents a good correlation between corneal and scleral IOP measurements in healthy subjects [12,13], so it has also been used to measure IOP during SL wear [2,14]. Nau et al. [14] reported that scleral IOP measurements made while wearing SLs were not accurate and, similar to Fogt et al. [2], observed that scleral IOP was approximately 6 mmHg higher than corneal IOP. Similarly, scleral IOP measurement with the rebound tonometer during SL wearing showed a poor correlation with corneal IOP [15]. These studies do not observe an increase in IOP during the wearing of SLs; however, they measure IOP at the sclera with tonometers that are calibrated to obtain corneal measurements [2,14,15].

Suction forces have been mentioned as one of the possible reasons for the increase in IOP [3,16,17]. This elevation would be caused by the loss of FR during the settling of the SL, which would increase the subatmospheric pressure under the lens; however, this is unlikely to occur [18]. Furthermore, studies evaluating suction forces during lens wear rule out their presence [5,19]. Another possibility is that the compression produced by the support SL area on the episcleral veins and adjacent structures, such as the iridocorneal angle (ICA) or Schlemm’s canal (SC), increases resistance to aqueous humor outflow [18,20]. The use of a SL with a larger diameter could decrease compression by distributing the weight over a wider bearing surface [14,21], compared to a smaller diameter lens, in which a greater displacement of intraocular fluid due to tangential flattening could increase IOP [14]. Larger diameter SLs [16,22] and lens fittings with a lower initial fluid reservoir (FR) in the central zone [16] were found to produce less settling. However, the validity of this hypothesis remains questionable, as several studies have not found a relationship between IOP and compression produced by SLs of different diameters [1,2].

SLs overlie several structures on which they can produce morphologic changes, including the cornea; lens wear longer than eight hours induced an incidence of oedema of about two percent [16,23,24,25], due to the central lens thickness and FR that make oxygen flow difficult [26]. However, this oedema is lower in incidence than the physiological oedema by around four percent, which occurs during sleep [24]. Studies that have analyzed the changes produced by SLs in the trabecular iris angle (TIA) have found no changes over four hours of wear [27] or after lens removal [1,27]. In the landing zone, the greatest compression occurs in the adjacent conjunctival/episcleral tissue, while in the scleral tissue, this compression is less than two percent [28]. Despite the fact that the changes in this tissue are small, the impact that SLs have on the SC has not been studied.

The optic nerve head has also been analyzed, searching for possible changes resulting from IOP increases after the use of SLs [8,29]. The Bruch’s membrane opening relative to the minimum ring amplitude (BMO-MRW), measured by OCT, is a parameter able to detect these changes with a robustness and sensitivity similar to the retinal nerve fiber layer (RNFL) analysis [30]. However, the results obtained are controversial; while some studies found a reduction in this parameter during six hours of SL wear [29], others did not find changes in the same time frame or after SL removal, as well as no relationship between BMO-MRW and IOP [8].

Previous studies have separately assessed the changes produced by SLs either in the different anterior segment structures or in IOP. The aim of this work was to evaluate, in a comprehensive and combined way, the short-term changes produced by SLs in the cornea, ICA, and SC structures and measurements with AS-OCT, as well as in IOP, including the structural parameters of the optic nerve head with OCT.

## 2. Materials and Methods

This longitudinal, prospective study was conducted in accordance with the Declaration of Helsinki and was approved by the Research Ethics Committee of the Principado de Asturias (protocol number 2020.490). All participants signed the informed consent form explaining the nature and objectives of the study. Thirty healthy subjects were recruited for this study. They underwent a complete ophthalmologic examination, including a detailed anamnesis, visual acuity, retinoscopy, noncycloplejic automated refraction, biomicroscopic examination of the anterior segment, fundus examination with a 90 D lens, Posner lens gonioscopy, Perkins applanation tonometry (PAT), and corneal topography. Exclusion criteria were previous refractive surgery, family history of glaucoma, medication that produces an increase in IOP (corticosteroids, anxiolotics, antidepressants, etc., cup/disc ratio > 0.5 or with eye-to-eye asymmetry > 0.2, refractive errors < −6 D, visible horizontal iris diameter (HVID) > 12.30 mm, ICA ≤ grade 3 (Shaffer scale) [31], synechiae or pigmentation grade 3+ or greater (Spaeth scale) [32] measured by gonioscopy, IOP ≥ 21 mmHg, and SL users. All study subjects were advised to avoid drinking coffee or caffeinated beverages for at least 24 h before the assessment.

In the study protocol, prior to the screening process, it was determined that the right eye would be fitted with the SL (the study eye), while the left eye remained without a lens (the control eye). The order of measurements was also established. First, the optic nerve head and SC parameters were measured with the Optovue. Next, ICA and CCT parameters were measured with the CASIA2. Finally, IOP was measured first with the TT, which was the least invasive tonometer, and then with the PAT. A period of time was left between the measurements of both tonometers to avoid bias in the measurements.

### 2.1. Scleral Contact Lenses

**Figure 1:**
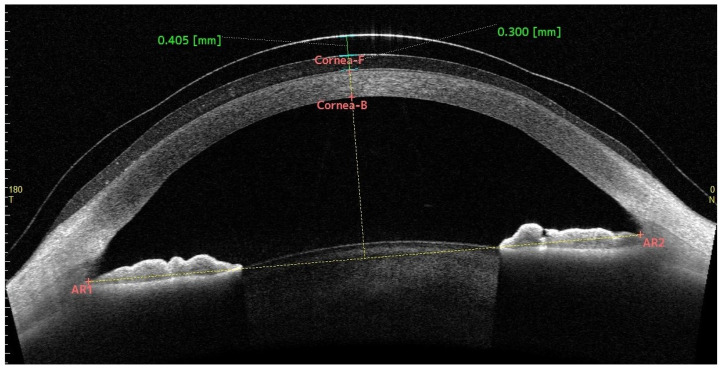
Lens thickness and fluid reservoir thickness measured with CASIA2 at the time of SL insertion (0 h).

A 15.8 mm diameter lens (L1) and a 16.8 mm diameter lens (L2) (Paflufocon B) (ICD Flexfit, Lenticon, Spain) were adapted from a trial box of 5 lenses of each diameter and 200 µm steps in sagittal height with a landing zone (steep +5). Lens fitting was performed according to the fitting guide provided by the manufacturer, starting from an initial fluid reservoir (FR) thickness between 250 and 400 µm (Figure 1). It was verified that the landing zone was aligned with the sclera in all patients and that there were no whitening and/or compression areas. All participants had the L1 inserted first. The investigator was instructed to exert minimal pressure during lens insertion and removal. Physiological saline 0.9% (Braun Medical S.A., Barcelona, Spain) was instilled into all lenses for application. The patient was instructed to avoid physical exertion and forced and prolonged palpebral closure during the 2 h of lens wear. A period of one week was left between the insertion of both SLs. 

### 2.2. Measurement of IOP

IOP measurements were performed with two tonometers. The Perkins MK2 (PAT) (Haag-Streig Holding, Harlow, UK) is a portable Goldmann applanation tonometer. It was used to test diurnal variations in IOP by taking 3 measurements in each eye between 08:00 and 10:00 a.m., as well as to measure IOP before insertion and after removal of the SL in the study eye and control eye. A drop of fluorescein sodium (2.5 mg) and oxybuprocaine hydrochloride (4 mg) (Colircusí Fluotest, Alcon Healthcare S.A., Barcelona, Spain) was instilled for measurements. The tonometer was calibrated every week, following the device’s manual. 

The Diaton transpalpebral tonometer (TT, Ryazan State Instrument-Making Enterprise, Ryazan, Russia) is based on a ballistic principle to determine IOP. It was used to measure IOP before insertion, during wear (0 h, 1 h, and 2 h), and after removal of the lens in the study eye and in the control eye. With the patients seated, they were asked to tilt their heads back and look straight ahead, forming a 45° angle with their line of gaze. Subsequently, the upper eyelid was slightly lifted, leaving the sclerocorneal limbus visible, and the tonometer was placed vertically to the eyelid, 1 mm behind the tarsus. When the device stops emitting sound, it means that it is ready to perform the measurement, which occurs automatically when the rod touches the eyelid. The tonometer takes several measurements and averages them.

### 2.3. Measurement of Iridocorneal Angle (ICA) and Schlemm Channel (SC) Parameters

**Figure 2:**
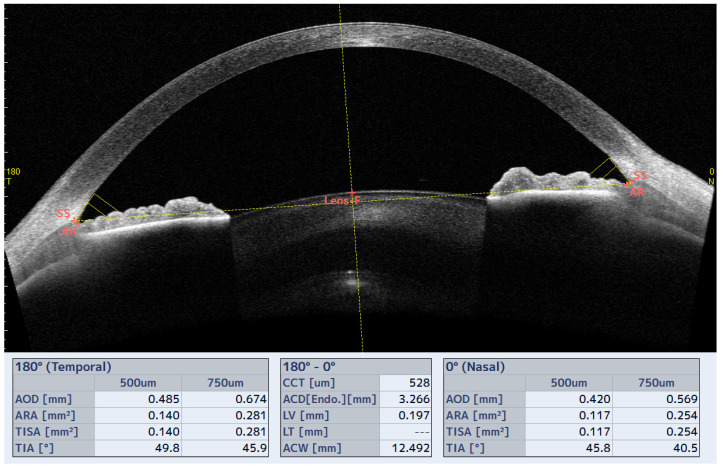
ICA parameters measured with CASIA2 in the horizontal axis (0–180°) before insertion of the SL.

The CASIA2 (Tomey, Nagoya, Japan) is a swept source OCT (SS-OCT) designed for anterior segment study (AS-OCT) that allows quantitative analysis of ICA and other anterior segment parameters. The ICA parameters measured were trabecular iris angle (TIA500), trabecular iris area (TISA500), angle opening distance (AOD500), and angle recess area (ARA500) at 500 µm from the scleral spur (SS) in the horizontal (0–180°) and vertical (90–270°) axes (Figure 2), in addition to the trabecular iris contact index (ITC index). Another parameter that was also measured was the central corneal thickness (CCT).

All these measurements were performed before lens insertion and after lens removal in the study eye, except for CCT, which was also performed in the control eye. This device was also used to measure parameters related to SLs, such as sagittal ocular height (SAG-OC) in the horizontal (0–180°) and vertical (90–270°) axes before lens insertion, lens thickness in these axes at the time of insertion (0 h), and central FR thickness during wear (0 h, 1 h, and 2 h) in these same axes (Figure 1). Finally, the mean value of the central FR thickness and lens thickness obtained in both axes was calculated. 

The Optovue RTVue 100 (Optovue, Fremont, CA, USA) is a spectral domain OCT (SD-OCT) incorporating CAM-L and CAM-S modules for anterior segment scanning. The CAM-S module, with a scan size of 2 mm × 2 mm, has higher resolution, so it was used to measure the length and area of the SC in the nasal and temporal regions. To perform the measurements in the same area, the patient was asked to maintain fixation on the central point shown by the device. At that time, the arrow shown by the device on the screen and indicating the location of the scan was placed above the limbus, leaving the same distance on both sides of the limbus, while the perpendicular straight line cutting the arrow was placed tangent to the limbus. The quality of the images was checked with the indicator provided by the device, and measurements were taken before insertion and after removal of the lens. 

### 2.4. Measurement of Optic Nerve Head Parameters

The Optovue RTVue (Optovue, Fremont, CA, USA) was also used to analyze the optic nerve head by removing the CAM-S module. In addition, RNFL thickness, neuroretinal ring area (NRA), and neuroretinal ring volume (NRV) were measured in the study eye and control eye before and after removal of the SL. 

### 2.5. Statistical Analysis

A sample size calculation was performed with statistical software Granmo version 7.12 (Institut Municipal d’investigacio’ Mèdica; Barcelona, Spain), considering mean IOP as the main variable. A risk of α = 0.05 and β = 0.20 was accepted in a bilateral test. Twenty-four subjects were necessary to detect a statistically significant difference greater than or equal to 3 mmHg with a standard deviation of 5 mmHg. A total of thirty-two subjects were recruited, and thirty finally completed all the tests. Statistical analysis was performed with SPSS^®^ for Windows (version 22.0; SPSS Inc.; Chicago, IL, USA). Three measurements of all parameters were performed, and the mean was calculated. 

The normality of the sample was assessed with the Saphiro–Wilk test. For the comparison of the mean values of the parameters studied before and after removing the lens and the correlation between the measurements, the repeated measures *t*-test and Pearson’s correlation test were used, respectively. The Wilcoxon test and Spearman’s correlation were applied when the sample did not have a normal distribution. Changes in all parameters analyzed were calculated as Δ equaling the mean value after lens removal minus the mean value before lens insertion. A multivariate linear regression model was employed to study the relationship between several variables. Intrasession repeatability of TT and PAT was calculated by within-subject standard deviation (Sw), which was used for within-subject precision (Sw × 1.96), repeatability (2.77 × Sw), and coefficient of variation (CV), defined as CV = Sw/mean × 100 [%]. The agreement in IOP measurements between the two tonometers was studied using Bland and Altman plots, where 95% of the difference or limits of agreement (LoA) were between 1.96 and the standard deviation (SD) of the mean difference. The degree of statistical significance was *p* < 0.05.

## 3. Results

Thirty-two subjects who visited the Instituto Oftalmológico Fernández-Vega between June 2021 and May 2022 participated in this study. Two of them did not complete the protocol because, at the end of the period wearing the first lens, they decided to drop out voluntarily. The descriptive characteristics of the sample are shown in Table 1.

### 3.1. Scleral Contact Lenses

The mean sagittal depth was 3940.00 ± 167.33 µm with L1 and 3980.00 ± 198.96 µm with L2 (*p* = 0.01). The mean thicknesses of L1 (399.62 ± 45.21 µm) and L2 (399.85 ± 50.35 µm) were similar (*p* = 0.89).

### 3.2. Central Fluid Reservoir

The initial central FR with L1 was 355.08 ± 85.81 µm, and with L2 it was 352.50 ± 74.56 µm (*p* = 0.89). After 2 h of wearing the SL, the central FR with L1 was reduced by 137.50 ± 40.97 µm, and with L2 by 117.50 ± 41.81 µm (*p* = 0.04).

### 3.3. Central Corneal Thickness

The initial CCT was 537.53 ± 34.10 µm with L1 and 545.63 ± 33.70 µm with L2 (*p* = 0.37). After 2 h of wearing the SL, there was an increase in CCT with L1 with respect to the initial value of 8.10 ± 4.21 µm (*p* < 0.01), representing an increase of 1.50%. After L2 wearing, the CCT increased by 9.17 ± 4.41 µm (*p* < 0.01), which is equivalent to an increase in CCT of 1.71%. 

In the control eye, no difference in initial CCT was observed with L1 (539.03 ± 28.04 µm) and with L2 (539.27 ± 23.61 µm) (*p* = 0.42). After 2 h, matching the removal of the SL in the study eye, the CCT did not undergo significant changes with L1 (−0.87 ± 6.53 µm) (*p* = 0.46) and L2 (0.70 ± 6.31 µm) (*p* = 0.55), representing a reduction of 0.16% and an increase of 0.13%, respectively. In the study eye and control eye, no differences were observed in the initial CCT with L1 (*p* = 0.46) and L2 (*p* = 0.53).

Using a multivariate linear regression model, it was tested whether the change in CCT in the study eye was related to lens thickness (LT) and initial FR (0 h), finding no relationship with the variables studied in L1 (constant = 2.32, LT = 0.02, FR = 0.00, R2 = 0.03, *p* = 0.66) and L2 (constant = 2.96, LT = 0.02, FR = 0.00, R2 = 0.07, *p* = 0.38).

### 3.4. Iridocorneal Angle and Schlemm’s Canal Parameters

**Figure 3:**
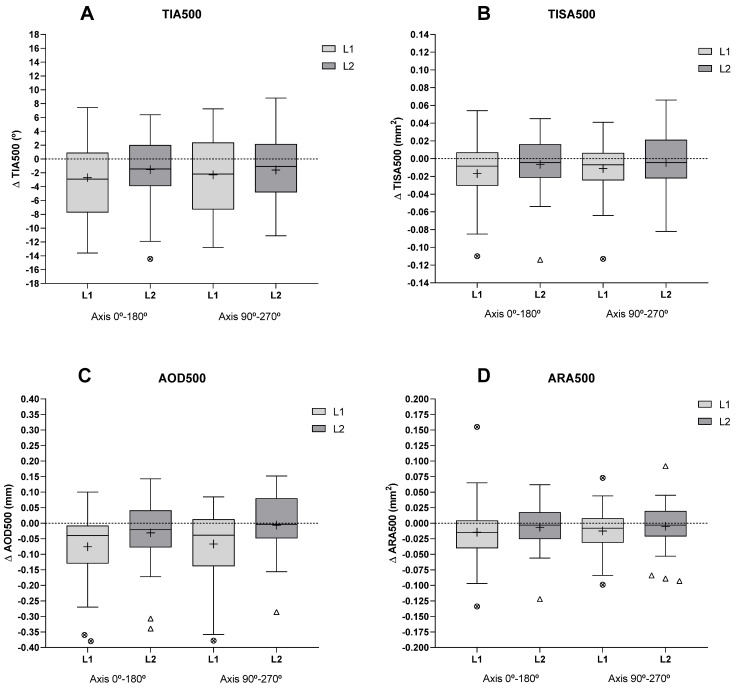
The box plot shows the change (Δ) in ICA parameters after L1 and L2 removals relative to pre-insertion values. (**A**) trabecular iris angle (TIA500); (**B**) trabecular iris area (TISA500); (**C**) angle opening distance (AOD500); (**D**) angle recess area (ARA500). The line inside the box represents the median, and the cross represents the mean. The lower and upper borders of the boxes represent the 25th and 75th percentiles, the lower and upper bars show the 10th and 90th percentiles, and the dots and triangles show the outliers for each lens. The dashed horizontal line indicates no change. Δ represents the difference between the values after and before lens wear (Negative values show a decrease and positive values an increase).

The ICA parameters (TIA500, TISA500, AOD500, and ARA500) in the axes 0–180° and 90–270° and the ITC analysis, as well as the SC (length and area of the SC in the nasal and temporal areas), showed no significant differences when comparing the values obtained before L1 and L2 insertion (*p* > 0.05). A decrease in ICA parameters was observed with L1 except in TISA500 and ARA500 (90–270°) (Figure 3) and in the ITC index. Similar to this parameter, no changes were observed with L2 (Table 2).

In contrast, SC area and length decreased in the nasal and temporal sectors after wearing L2. With L1, a decrease in SC area was only observed in the temporal sector (Table 2).

The relationship between changes in the ICA and SC parameters and changes in IOP with L1 and L2 in the study eye was studied. A positive correlation was observed between the Δ IOP PAT and the Δ ITC with L1 (ρ = 0.44, *p* = 0.02) and L2 (ρ = 0.45, *p* = 0.01). The rest of the results can be seen in Appendix A. 

### 3.5. Intraocular Pressure (IOP)

In the study eye, no differences in IOP PAT were observed before L1 and L2 insertion (*p* = 0.85). Before L1 insertion, IOP PAT in the study eye and control eye were not different (*p* = 0.75). However, with L2, significant differences in IOP PAT were obtained (*p* = 0.01). In the study eye, after both lenses were removed, IOP increased, while in the control eye it decreased (Table 3).

**Figure 4:**
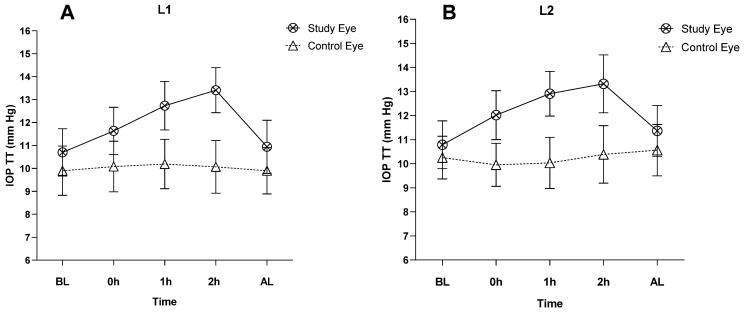
IOP with TT before, during, and after wearing the SL in the study eye and the control eye. (**A**) L1 (15.8 mm lens); (**B**) L2 (16.8 mm lens).

Before L1 insertion, IOP TT was different in the study eye and control eye (*p* = 0.08), while with L2, it was not different (*p* = 0.44). In the study eye, IOP TT was not different before L1 and L2 insertion (*p* = 0.90). In this eye, IOP TT increased significantly during L1 and L2 wearing, while in the control eye, no changes were observed during the same time (Table 4 and Figure 4). 

In the study eye, ΔCCT was not related to ΔIOP PAT (r = 0.01; *p* = 0.96) nor to ΔIOP TT (r = −0.15; *p* = 0.44) after wearing L1. The ΔCCT observed with L2 was also unrelated to ΔIOP PAT (r = −0.26; *p* = 0.17) and ΔIOP TT (r = 0.31; *p* = 0.09).

In the control eye, there was also no relationship between ΔCCT and ΔIOP PAT (ρ = −0.12; *p* = 0.54) and ΔIOP TT (ρ = 0.23; *p* = 0.22) with L1 wear, nor with ΔIOP PAT (ρ = −0.11; *p* = 0.56) and ΔIOP TT (ρ = −0.18; *p* = 0.34) with L2 wear.

These results showed individual variability in IOP values. IOP TT in the study eye after wearing L1 decreased in 11 patients (36.67%), showed no change in 5 patients (16.67%), and increased in 15 patients (50.00%), with one of them increasing by 4 mmHg. After wearing L2, IOP TT decreased in 7 patients (23.33%), showed no change in 5 patients (16.67%), and increased in 18 patients (60.00%), with one of them increasing up to 3.33 mmHg. 

However, while wearing the lens, IOP TT with L1 increased in 28 patients (93.33%). Of these 28 patients, 18 (60.00%) had an increase ≥ 2 mmHg, and 4 (13.33%) had an increase ≥ 5 mmHg. With L2, IOP TT increased in 26 patients (86.67%), with an increase ≥ 2 mmHg in 19 patients (63.33%) and ≥5 mmHg in 3 patients (10.00%).

**Figure 5:**
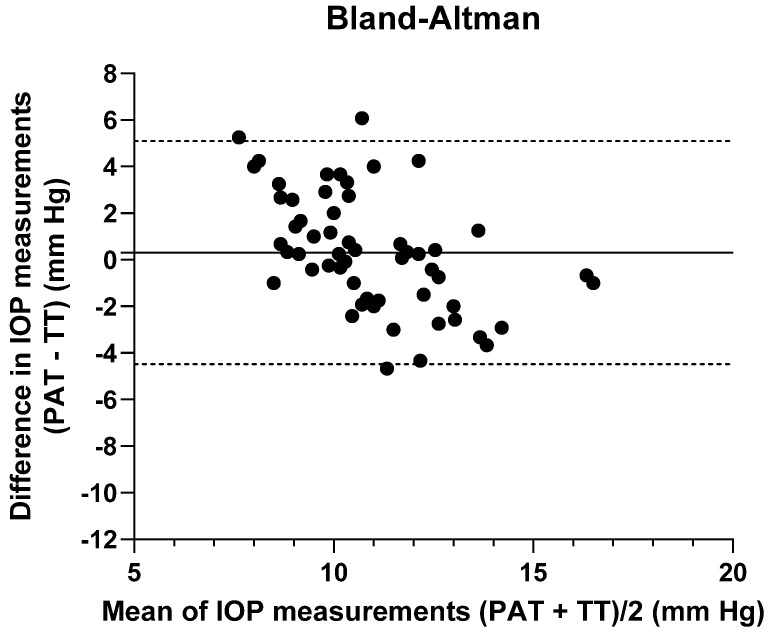
Bland and Altman plot of IOP agreement between TT and PAT. Mean difference of 0.31 ± 2.11 mmHg; limits of agreement (LoA) from −4.48 to 5.08.

The comparison between the mean IOP values between TT and PAT was performed with the 60 measurements obtained before the insertion of both lenses in the study eye (30 measurements from L1 and 30 from L2). The IOP PAT (11.05 ± 1.63 mmHg) was slightly higher than the IOP TT (10.74 ± 2.71 mmHg), but this difference was not significant (*p* = 0.44). The correlation between the measurements of both instruments was moderate (ρ = 0.50, *p* = 0.01). The repeatability of PAT and TT was 4.56 and 7.57, respectively. The CV was higher with the TT (25.43%) than with the PAT (14.90%). The agreement between PAT and TT was 0.31 ± 2.11 mmHg (LoA from −4.48 to 5.08) (Figure 5). 

### 3.6. Optic Nerve Head Parameters

No significant differences were observed when comparing RNFL thickness (*p* = 0.38), NRA (*p* = 0.50), and NRV (*p* = 0.19) values with OCT before L1 and L2 insertion in the study eye. We also compared the values before L1 insertion in the study eye and control eye without finding differences in RNFL thickness (*p* = 0.16), NRA (*p* = 0.52), or NRV (*p* = 0.27) values. Similar results were found when comparing RNFL thickness (*p* = 0.12), NRA (*p* = 0.85), and NRV (*p* = 0.51) values in the study eye and the control eye with L2. 

RNFL thickness, NRA, and NRV values also did not experience significant changes after removal of L1 and L2 in the study eye and control eye (Table 5).

The relationship between changes produced in optic nerve head parameters and IOP was also evaluated. In the study eye, an inverse correlation was observed between ΔIOP TT and ΔNRA (ρ = −0.37, *p* = 0.04) with L1, while in the control eye, ΔIOP TT and ΔRNFL (ρ = −0.41, *p* = 0.02) with L1 and ΔIOP PAT and ΔNRV (ρ = −0.46, *p* = 0.01) with L2 were also related (Appendix B).

## 4. Discussion

In the present study, a reduction in FR of 137.50 µm with L1 and 117.50 µm with L2 was found after two hours of wear. Other studies have reported reductions in FR between 76 and 133 µm after six and eight hours of wear [22,33,34]. The greatest decrease occurs mainly in the first two to four hours after insertion [22]. Moreover, this decrease in FR varies with lens design and diameter [16,22], being lower in larger SLs. This is probably because the larger SL rests further away from the limbus, where the episclera is thicker [35]. Additionally, due to the changes in FR during the two hours of L1 and L2 wear, there was an increase of 8.10 and 9.17 µm, respectively, in CCT. This increase was greater than the ratio established by Munford et al. [36] of 1 µm per hour with a high-permeability SL (DK 120) and is consistent with the increase observed in some studies that evaluated SLs during eight hours of wearing [16,23]. However, the oedema observed in this study (<2%) is lower than the physiological oedema that occurs during sleep (4%) [24]. The cause of this oedema is insufficient oxygen supply to the cornea, known as hypoxia, due to the thickness of the lens [37]. In the case of SLs, in addition to the thickness of the lens itself, the thickness of the FR also plays a role. However, the multivariate model showed that the increase in CCT with lens wear was not related to lens thickness or initial FR. This may be due to the DK of the lens, which, in preventing hypoxia, is more important than the thickness of the FR [24].

The studies that evaluated the influence of the SLs on the AIC did not observe any changes [1,27]. However, these studies measure AIC globally [1] or in the temporal area [27], so changes in a specific meridian may go undetected. In addition, AIC analysis is performed with a Scheimpflug camera system, which provides limited resolution of the AIC [38]. In this study, TIA500, TISA500, AOD500, and ARA500 were analyzed in the principal axes, 0–180° and 90–270°, with CASIA 2, an AS-OCT that allows visualization of the AIC at a higher resolution [39]. A decrease in all parameters was found in the horizontal meridian after the smallest lens diameter (L1). In this meridian, the sclera becomes flatter, and thus the SL produces a higher mechanical pressure in this area [40], which may cause changes in adjacent structures such as the ICA and subsequently in its parameters. In the vertical meridian, the decrease in TIA500 and AOD500 may be related to greater compression of the SL on the conjunctiva in the upper zone due to the mechanical pressure exerted by the eyelids [28]. Furthermore, unlike the horizontal meridian, no changes in parameters such as TISA500 and ARA500, which represent areas, were observed in this meridian. This may be because these parameters need more compression to observe significant changes compared to TIA500 and AOD500, which do not represent an area. The ITC was the only ICA parameter studied globally for which no change was observed. It has been seen that TIA is less than 23.2° if there is an iridotrabecular contact [41]. However, the highest reduction in TIA after SL wear, regardless of axis, was −2.72° with L1 and −1.60° with L2, resulting in a final value of 44.80° and 44.86°, respectively. These values are considerably higher than the limit established by Fernandez-Vigo et al. [41], so the relevance of the observed changes is limited. A similar situation occurred with AOD, which is a standardized parameter in the assessment of angular opening. In patients with angular closure, the value of this parameter was 0.2 mm [42]. However, this value is again considerably lower compared with that obtained in this study after wearing both SCLs (L1 = 0.49 mm and L2 = 0.52 mm).

In the SC, the area and length were reduced in the horizontal meridian after wearing the largest diameter lens (L2). The SC is located deeply in the corneoscleral tissue, covering more than two-thirds of its thickness [43]. The compression produced by the SL landing zone in this tissue is small (2% of its thickness) compared to 30% in the conjunctival/episcleral tissue [28]. However, the SC is not a rigid structure; it collapses (invisible) with increasing IOP [44]. It has been observed that the CS area decreased by 30% with an IOP increase of 23.2 mmHg [45]. In this study, although the CS area decreased by 18.07% in the nasal zone and 15.02% in the temporal zone after wearing L2, the 0.58 mmHg increase in IOP with the TT and 0.23 mmHg with the PAT was considerably less than that observed by Kageman et al. [45]. Furthermore, IOP with both tonometers was not related to CS area or CS length, which also decreased in both areas after L2 use and is a more reproducible parameter [46]. Therefore, an increase in IOP due to CS compression with L2 in healthy patients seems improbable. However, this compression may have different implications for IOP in patients with glaucoma, in whom the CS area is smaller [46,47].

Changes in the CS with L2 (larger diameter) are possibly related to a slightly posterior location of this structure with respect to the AIC, where changes are observed with L1 (smaller diameter), as the size of the supporting area is the same for both LSs (1.5 mm).

During the two hours of wearing both SLs, a similar increase in IOP measured with the TT was observed in the study eye, while the control eye showed little change. A slightly higher increase (between 4.4 and 5.5 mmHg) was also observed by several authors who measured IOP with TT during one and four hours of wearing SLs of different sizes [1,2]. However, Fogt et al. [2] observed a rapid increase in IOP of 4.4 mmHg with the 15.2 mm lens and 5.0 mmHg with the 18.2 mm lens at the time of insertion, which persisted during lens wear. The decrease in the elasticity of the tissue adjacent to the lens landing zone in the superior sector, where the compression is greatest and the TT measurements are made, was attributed as a possible cause of the increase in IOP with this tonometer during lens wear [2]. Possibly, this fact occurs in this study because, despite being lower, a rapid increase was observed at the time of insertion (L1 = 0.93 mmHg, L2 = 1.23 mmHg). However, this different IOP behavior at the time of insertion in both studies may be due to the different tonicity of the eyelids of the sample tested as well as the characteristics of the scleral tissue. In this study, IOP was also measured with the PAT, with a slight increase in the study eye and a decrease in the control eye, although changes in IOP were not related to changes in CCT. The PAT measurements are comparable to the GAT [48] that was used in several studies to measure IOP after removal of SLs in patients with keratoconus and ocular surface disease [4,6,49]. In one of them, Shanhazi et al. [49] did not find an association between changes in IOP and CCT caused by wearing SLs. In this study, the relationship between changes in IOP and ICA was also studied as one of the possible hypotheses leading to an increase in IOP. A positive correlation was only observed between the ΔIOP PAT and the ΔITC index after wearing both SLs, although this finding is of limited clinical importance. According to some reports with the previous version of the AS-OCT platform used in the present study (CASIA SS-1000, Tomey, Nagoya, Japan), results greater than 35% for the ITC index provide good diagnostic ability for angle closure [50], which can also be associated with high IOP [51]. However, in this study, the ITC index before insertion of both SLs was <3.5%, and it increased by 1.28% with L1 and 1.52% with L2. As there was hardly any relationship between changes in IOP, ICA, and SC with SL wearing in this study with healthy subjects, it seems unlikely that an IOP increase could be due to changes in these structures. By contrast, authors think that IOP changes might be related to the degree of scleral applanation (or indentation) associated with the greater volume of displaced intraocular fluid [18]. However, Xu et al. [52] established cut-off points that, except for TIA500 (15.16°), TISA500 (0.046 mm^2^), AOD500 (0.104 mm), and ARA500 (0.047 mm^2^), were related to IOP. Although the study was conducted in the Asian population, it cannot be excluded that the changes produced by SL wear in subjects with AIC parameters close to these values may lead to an increase in IOP. 

Before insertion of both SLs, IOP PAT was slightly higher than IOP TT (0.31 mmHg), with moderate correlation and agreement between tonometers. By contrast, Formisano et al. [4] observed a consistent difference in IOP values (around 5 mmHg) between these tonometers before and after removal of SLs in patients with keratoconus. However, most studies in healthy and keratoconus patients have shown a poor/moderate correlation and agreement [9,10,11,53]. PAT showed lower CV and repeatability, similar to other studies [54,55]. However, TT is accurate when IOP measurements with GAT are between 11 and 21 mmHg [56]. In this range, according to the instructions provided by the manufacturer, the error of TT is 2 mmHg. However, the increase in IOP at two hours of wear with both lenses (L1 = 2.55 mmHg and L2 = 2.53 mmHg) was greater than the tonometer error, which would indicate that there is an increase in IOP during this time. Furthermore, the percentage of subjects who had an increase in IOP ≥ 2 mmHg during both SL wear was at least 60%, but similar to other studies, the intersubject variability was high [1,5].

RFNL thickness and optic nerve head parameters were also studied to indirectly determine the possible consequences of the potential IOP increase during SL wear. Other works studied changes in BMO-MRW under the same conditions, with different results [8,29]. This parameter is associated with an increase in IOP [30], and its position is susceptible to changes in IOP [57]. Therefore, it is possible that the difference in the IOP value and/or the position of the BMO-MRW between the two studies is the reason for the discrepancy in the results. However, no relationship between BMO-MRW and IOP has been observed with SLs [8]. In this study, changes found in RNFL thickness, NRA, and NRV values with the wearing of SLs were minimal or not clinically significant. Furthermore, most of the changes in these parameters are not related to changes in IOP with TT and PAT in the study eye. Only a positive relationship is observed between changes in NRA and IOP with TT after wearing L1. However, RFNL thickness, which is a parameter sensitive to IOP fluctuations [58], has also been related to changes in IOP TT in the L1 control eye. Similarly, the observed changes in NRV, which were also related to IOP changes with PAT in the L2 control eye, were of the same magnitude as those observed in the study eye after wearing L2. In addition, the changes observed in RFNL thickness and NRV after wearing both SLs are similar to the fluctuations shown by these parameters during the day [59,60]. Therefore, it is most likely that these observed changes in optic nerve head parameters after wearing both SLs are related to fluctuations in the eye itself and not to changes in IOP. However, these changes could be relevant in patients with glaucomatous damage, where small fluctuations in IOP produce changes in RFNL thickness that are predictive of glaucoma [58].

One of the limitations of this study was that, when examining the AIC and SC with different AS-OCT, the distance of these structures to the center of the eye and the centration of the lens were not taken into account. This may result in the decentration of the smaller lens resting in the area where the larger diameter lens does, and conversely, this is one of the possible reasons for the reduction in the SC area with L1. Therefore, it cannot be categorically said that the changes in the different structures are due to a specific lens size.

In the future, it would be important to establish a standard method for measuring IOP with SLs. In addition, it would be interesting to assess the changes produced by these types of contact lenses in ICA and SC in subjects with different angle widths, higher IOPs, and/or susceptibility to developing glaucomatous disease, as well as optic nerve head monitoring.

## 5. Conclusions

In the short term, SLs resulted in a slight increase in IOP as well as small changes in CCT, ICA, and SC, although they do not seem to be clinically relevant in healthy subjects. TT showed a moderate concordance with PAT; however, their measurements are not interchangeable in SL wearers.

## Figures and Tables

**Table 1:** Sample descriptive characteristics.

Characteristics	Value
Participants (*n*)	30
Sex (male/female)	14/16
CL wearers (yes/no)	11/19
Age (years)	28.97 ± 5.62
SAG-OC (0–180°) (µm)	3751.80 ± 194.09
SAG-OC (90–270°) (µm)	3758.47 ± 189.50
HVID (mm)	12.09 ± 0.16
SE (D)	−1.18 ± 1.39

CL = contact lenses; SAG-OC = sagittal ocular height; HVID = horizontal visible iris diameter; SE = spherical equivalent. Data are expressed as mean ± SD.

**Table 2:** Changes in ICA and SC parameters after wearing SLs.

		Before Lens (Mean ± SD)	After Lens (Mean ± SD)	Δ (Mean ± SD)	*p*
ICA parameters					
TIA500 0–180° (°)	L1	47.55 ± 14.19	44.80 ± 12.14	−2.72 ± 5.33	0.01 *
	L2	47.90 ± 13.74	46.43 ± 14.17	−1.54 ± 4.86	0.09
TIA500 90–270° (°)	L1	46.71 ± 12.76	44.10 ± 12.50	−2.30 ± 5.26	0.02 *
	L2	46.46 ± 12.75	44.86 ± 12.59	−1.60 ± 5.24	0.11
TISA500 0–180° (mm^2^)	L1	0.21 ± 0.08	0.20 ± 0.08	−0.02 ± 0.04	0.01 *
	L2	0.21 ± 0.08	0.20 ± 0.08	−0.01 ± 0.03	0.38
TISA500 90–270° (mm^2^)	L1	0.20 ± 0.08	0.19 ± 0.07	−0.01 ± 0.03	0.06
	L2	0.20 ± 0.07	0.19 ± 0.07	0.00 ± 0.04	0.43
AOD500 0–180° (mm)	L1	0.61 ± 0.24	0.53 ± 0.24	−0.08 ± 0.12	<0.01 *
	L2	0.59 ± 0.24	0.56 ± 0.24	−0.03 ± 0.11	0.14
AOD500 90–270° (mm)	L1	0.56 ± 0.23	0.49 ± 0.22	−0.07 ± 0.12	0.00 *
	L2	0.53 ± 0.18	0.52 ± 0.19	−0.01 ± 0.10	0.72
ARA500 0–180° (mm^2^)	L1	0.23 ± 0.09	0.22 ± 0.09	−0.02 ± 0.05	0.04 *
	L2	0.22 ± 0.09	0.22 ± 0.08	−0.01 ± 0.04	0.47
ARA500 90–270° (mm^2^)	L1	0.21 ± 0.08	0.20 ± 0.08	−0.01 ± 0.04	0.09
	L2	0.21 ± 0.07	0.21 ± 0.07	−0.01 ± 0.04	0.61
ITC index (%)	L1	3.26 ± 9.00	4.53 ± 12.08	1.28 ± 4.53	0.19
	L2	2.62 ± 7.92	4.03 ± 9.83	1.52 ± 7.99	0.15
SC parameters					
SC Nasal Lenght (µm)	L1	260.56 ± 51.87	252.91 ± 43.05	−8.66 ± 50.57	0.36
	L2	261.51 ± 47.22	235.56 ± 37.96	−25.96 ± 46.60	0.01 *
SC Temp Lenght (µm)	L1	269.51 ± 54.99	261.56 ± 57.87	−7.96 ± 42.96	0.32
	L2	275.71 ± 54.22	249.21 ± 55.76	−26.50 ± 45.36	<0.01 *
SC Nasal Area (µm^2^)	L1	5411.11 ± 1603.84	4977.78 ± 1637.52	−433.33 ± 1411.91	0.10
	L2	5777.78 ± 1967.81	4733.34 ± 1450.06	−1044.44 ± 1852.27	<0.01 *
SC Temp. Area (µm^2^)	L1	6022.22 ± 1909.71	5111.11 ± 1749.29	−911.11 ± 1324.49	<0.01 *
	L2	5944.45 ± 1830.11	5088.89 ± 1623.42	−855.56 ± 1601.45	0.01 *

ICA = iridocorneal angle; TIA500 = trabecular iris angle; AOD500 = angle opening distance; ARA500 = angle recess area; TISA500 = iris-trabecular area; ITC = iris-trabecular contact; SC = Schlemm’s channel; Δ = the difference between the values after and before lens wear (Negative values show a decrease and positive values an increase). The asterisk (*) in the table indicates significant differences (*p* < 0.05).

**Table 3:** Measurement of IOP with the PAT before and after wearing SLs in the study eye and the control eye.

		Study Eye (Right Eye)			Control Eye (Left Eye)		
	Time	IOP (Mean ± SD)	Δ IOP (Mean ± SD)	*p*	IOP (Mean ± SD)	Δ IOP (Mean ± SD)	*p*
L1	BL	11.09 ± 1.66	-	-	11.14 ± 1.75	-	-
	AL	11.54 ± 2.82	0.46 ± 1.07	0.02 *	11.09 ± 1.66	−0.06 ± 0.77	0.87
L2	BL	11.02 ± 1.63	-	-	11.61 ± 1.79	-	-
	AL	11.25 ± 1.86	0.23 ± 1.16	0.44	11.29 ± 1.86	−0.32 ± 1.16	0.04 *

BL = before the lens; AL = after removing the lens. Δ = the difference between the values after and before lens wear (Negative values show a decrease and positive values an increase). The asterisk (*) in the table indicates significant differences (*p* < 0.05).

**Table 4:** Measurement of IOP with the TT before, during, and after wearing SLs in the study eye and the control eye.

		Study Eye (Right Eye)			Control Eye (Left Eye)		
	Time	IOP (Mean ± SD)	ΔIOP (Mean ± SD)	*p*	IOP (Mean ± SD)	Δ IOP (Mean ± SD)	*p*
L1	BL	10.70 ± 2.80	-	-	9.90 ± 2.92	-	-
	0 h	11.63 ± 2.82	0.93 ± 2.34	0.04	10.08 ± 3.00	0.18 ± 1.32	1.00
	1 h	12.73 ± 2.87	2.03 ± 1.79	<0.01 *	10.19 ± 2.93	0.29 ± 1.19	1.00
	2 h	13.25 ± 3.26	2.55 ± 2.04	<0.01 *	10.07 ± 3.13	0.17 ± 1.68	1.00
	AL	10.95 ± 3.15	0.24 ± 1.69	1.00	9.90 ± 2.76	0.00 ± 1.31	1.00
L2	BL	10.79 ± 2.66	-	-	10.56 ± 2.38	-	-
	0 h	12.02 ± 2.73	1.23 ± 1.49	<0.01 *	9.96 ± 2.40	−0.30 ± 1.35	1.00
	1 h	12.91 ± 2.49	2.12 ± 1.99	<0.01 *	10.03 ± 2.85	−0.22 ± 1.86	1.00
	2 h	13.32 ± 3.22	2.53 ± 2.22	<0.01 *	10.39 ± 3.20	0.13 ± 1.89	1.00
	AL	11.37 ± 2.84	0.58 ± 1.38	0.29	10.57 ± 2.85	0.31 ± 1.32	1.00

BL = before lens fitting; 0 h = immediately after lens fitting; 1 h = one hour after fitting; 2 h = two hours after fitting; AL = after lens removal. Δ = the difference between the several time intervals with respect to BL (Negative values show a decrease and positive values an increase). The asterisk (*) in the table indicates significant differences (*p* < 0.05).

**Table 5:** Changes in the retinal nerve fiber layer (RFNL) and optic nerve head parameters while wearing L1 and L2 in the study and control eyes.

		Study Eye (Right Eye)				Control Eye (Left Eye)			
		Before (Mean ± SD)	After (Mean ± SD)	Δ (Mean ± SD)	*p*	Before (Mean ± SD)	After (Mean ± SD)	Δ (Mean ± SD)	*p*
L1	RNFLt (µm)	107.76 ± 10.60	108.04 ± 10.25	0.28 ± 2.32	0.51	109.02 ± 9.90	109.04 ± 9.80	0.20 ± 2.06	0.96
	NRA (mm^2^)	1.67 ± 0.39	1.63 ± 0.42	−0.04 ± 0.12	0.09	1.64 ± 0.34	1.65 ± 0.35	0.01 ± 0.15	0.65
	NRV (mm^3^)	0.24 ± 0.08	0.23 ± 0.09	−0.01 ± 0.03	0.34	0.24 ± 0.11	0.24 ± 0.11	0.00 ± 0.03	0.66
L2	RNFL (µm)	106.95 ± 9.50	106.70 ± 10.64	−0.26 ± 2.82	0.62	108.23 ± 10.35	107.93 ± 9.93	−0.30 ± 3.48	0.64
	NRA (mm^2^)	1.65 ± 0.41	1.64 ± 0.42	−0.01 ± 0.12	0.71	1.65 ± 0.34	1.66 ± 0.34	0.01 ± 0.11	0.93
	NRV (mm^3^)	0.23 ± 0.09	0.23 ± 0.09	0.00 ± 0.02	0.45	0.23 ± 0.10	0.24 ± 0.11	0.01 ± 0.02	0.15

RNFLt = retinal nerve fiber layer thickness; NRA = neuroretinal rim area; NRV = neuroretinal rim volume. Δ = the difference between the values after and before lens wearing (Negative values show a decrease and positive values an increase).

## Data Availability

All the obtained data used to support the findings of this study are available from the corresponding author upon reasonable request.

## Appendix A

**Table A1:** Correlation between the changes in IOP and ICA parameters after SL wearing.

		ΔIOP TT		ΔIOP PAT	
	Lens	Correlation	*p*	Correlation	*p*
ICA parameters					
ΔTIA500 0–180° axis (°)	L1	0.19	0.31	−0.23	0.22
	L2	−0.27	0.21	0.03	0.89
ΔTIA500 90–270° axis (°)	L1	0.24	0.21	−0.26	0.17
	L2	0.02	0.94	0.19	0.32
ΔTISA500 0–180° axis (mm^2^)	L1	0.21	0.24	−0.21	0.27
	L2	−0.04	0.82	0.05	0.81
ΔTISA500 90–270° axis (mm^2^)	L1	0.22	0.25	0.05	0.79
	L2	−0.11	0.57	0.22	0.25
ΔAOD500 0–180° axis (mm)	L1	0.32	0.09	0.08	0.69
	L2	0.13	0.50	0.05	0.81
ΔAOD500 90–270° axis (mm)	L1	0.29	0.12	0.15	0.42
	L2	−0.04	0.83	0.15	0.43
ΔARA500 0–180° axis (mm^2^)	L1	0.14	0.46	−0.12	0.52
	L2	0.10	0.59	0.08	0.66
ΔARA500 90–270° axis (mm^2^)	L1	0.10	0.59	−0.09	0.64
	L2	−0.10	0.59	0.04	0.85
ΔITC index (%)	L1	0.08	0.67	0.44	0.02 *
	L2	0.09	0.64	0.45	0.01 *
SC parameters					
SC Nasal Lenght (µm)	L1	0.07	0.71	0.05	0.78
	L2	0.26	0.16	0.16	0.42
SC Temp. Lenght (µm)	L1	0.29	0.12	0.19	0.32
	L2	−0.11	0.58	0.05	0.81
SC Nasal Área (µm^2^)	L1	−0.19	0.31	0.24	0.21
	L2	0.26	0.17	0.14	0.45
SC Temp. Área (µm^2^)	L1	0.00	1.00	−0.03	0.87
	L2	−0.07	0.71	0.03	0.88

IOP TT = Intraocular pressure with transpalpebral tonometry. IOP PAT = Intraocular pressure with perkins tonometry applanation; TIA500 = Trabecular iris angle; TISA500 = Trabecular iris area; AOD500 = Angle opening distance; ARA500 = Angle recess area; SC = Schlemm’s channel; Δ = represents the change obtained from the difference of the value when removing the SL minus the value before inserting the lenses. The asterisk (*) in the table indicates significant differences (*p* < 0.05).

## Appendix B

**Table A2:** Correlation between changes in IOP and optic nerve head parameters after wearing SL.

			Study Eye (Right Eye)		Control Eye (Left Eye)	
	Parameters	Lens	Correlation	*p*	Correlation	*p*
Δ IOP TT (mmHg)	ΔRNFL (µm)	L1	−0.10	0.60	−0.41	0.02 *
		L2	0.04	0.85	0.15	0.42
	ΔNRA (µm^2^)	L1	−0.37	0.04 *	0.15	0.44
		L2	0.00	0.99	0.25	0.19
	ΔNRV (µm^3^)	L1	−0.34	0.07	0.07	0.73
		L2	0.00	0.99	0.25	0.18
Δ IOP PAT (mmHg)	ΔRNFL (µm)	L1	0.33	0.07	−0.34	0.06
		L2	0.05	0.81	−0.10	0.58
	ΔNRA (µm^2^)	L1	0.11	0.56	0.19	0.30
		L2	0.13	0.50	−0.35	0.06
	ΔNRV (µm^3^)	L1	0.12	0.52	0.25	0.19
		L2	−0.12	0.52	−0.46	0.01 *

IOP TT = Intraocular pressure with transpalpebral tonometry. IOP PAT = Intraocular pressure with perkins tonometry applanation; RNFL = Retinal nerve fibre layer; NRA = Neuroretinal rim area; NRV = Neuroretinal rim volume. Δ = represents the change obtained from the difference of the value when removing the SL minus the value before inserting the lenses. The asterisk (*) in the table indicates significant differences (*p* < 0.05).
